# The Downward Floral Orientation in *Polygonatum cyrtonema* Enhances Pollination Efficiency and Reproductive Fitness

**DOI:** 10.1002/ece3.73221

**Published:** 2026-03-08

**Authors:** Ju Tang, Deng‐fei Li, Xiang‐xiang Ge, Yu‐jie Xu, Jian‐wen Shao

**Affiliations:** ^1^ College of Life Sciences Anhui Normal University Wuhu Anhui China; ^2^ The Anhui Provincial Key Laboratory of Biodiversity Conservation and Ecological Security in the Yangtze River Basin Anhui Normal University Wuhu China; ^3^ Key Laboratory of Southwest China Wildlife Resources Conservation (Ministry of Education) China West Normal University Nanchong China

**Keywords:** floral orientation, pollen transfer, *Polygonatum cyrtonema*, reproductive fitness

## Abstract

Floral orientation as a key floral trait for understanding how plants integrate biotic and abiotic selective pressures. *Polygonatum cyrtonema* is an economically significant medicinal species. The adaptive significance of its downward floral orientation remains poorly understood. We conducted manipulation experiments to explore the visiting time per flower, visitation rate and the pollination efficiency of bumble bees and honey bees between downward flowers and artificially reoriented upward flowers. We also assessed pollen viability and stigma receptivity under both sunlight and rainwater exposure in the two orientations. Furthermore, we quantified reproductive fitness components to compare the fruit set, seed set, and the qualities of fruit/seed between two orientation flowers. Our results showed that the downward orientation enhances reproductive success through integrated biotic and abiotic factors. Downward flowers attracted more effective bumble bee pollinators, resulting in higher visitation rates, longer visiting time, and higher pollen transfer efficiency compared to upward flowers. While the upward floral orientation increased the visitation of ineffective visitors, such as honey bees and other syrphid flies. Simultaneously, the downward orientation flowers provided protection from solar radiation and rainwater, maintaining higher pollen viability, and stigma receptivity while reducing pollen loss. Although the fruit set per plant showed no significant difference between orientations, downward flowers developed significantly larger fruits with greater fresh mass and fruit size. Both the seed number and the seed set per fruit were significantly higher in downward flowers than upward ones. These results collectively support the pollinator attraction, pollinator filtering and abiotic protection hypothesis, demonstrating that the downward orientation in *P. cyrtonema* is shaped by both biotic and abiotic selection.

## Graphical abstract

We reveal that the downward floral orientation in *Polygonatum cyrtonema* functions as a key floral trait, increasing visitation by effective bumble bees and reducing interference from inefficient insects, while also shielding reproductive tissues from rain and solar radiation. Floral orientation as an adaptive trait shaped by both biotic and abiotic selection, ultimately promoting higher seed set and fruit quality, and highlighting its role in improving reproductive success under natural conditions.

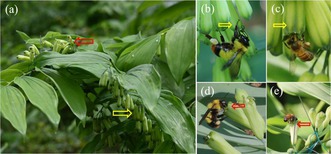

## 1 Introduction

Floral orientation is recognized as a critical functional trait influencing plant reproductive success through multiple ecological mechanisms (Hodges et al. 2002; Fenster et al. 2004). This trait reflects complex adaptations to both biotic and abiotic selective pressures. Biotic factors are mainly related to pollinators, such as differences in pollination efficiency and visitation frequency among pollinator groups (Wang, Xiao, et al. 2014; LoPresti et al. 2020; Prokop et al. 2023; Ge et al. 2025). Major abiotic factors involve environmental factors like solar radiation (UV‐B) and rainwater, which can directly affect flower development and reproductive fitness (Huang et al. 2002; Wang et al. 2010; Creux et al. 2021). Thus, floral orientation plays a particularly significant role in mediating both plant‐pollinator interactions and plant‐environment interactions.

Given that approximately 90% of flowering plants rely on animal pollinators for sexual reproduction (Tong et al. 2023), the ecological significance of floral orientation has been increasingly documented across diverse species, with strong evidence supporting its role in pollinator‐mediated selection (Fenster et al. 2009; Ushimaru et al. 2009; Wang, Tie, et al. 2014; Wang, Xiao, et al. 2014). Substantial empirical evidence indicates that floral orientation directly influences pollinator visitation frequency and foraging behavior across both specialized and generalized pollination systems (Fulton and Hodges 1999; Tadey and Aizen 2001; Ushimaru and Hyodo 2005; Wang, Tie, et al. 2014; Wang, Xiao, et al. 2014; Nevard and Vallejo‐Marín 2022). Critically, floral orientation affects the precision of pollen transfer (Prokop et al. 2023). For instance, a downward orientation can enhance pollen export efficiency by ensuring better contact between anthers and specific parts of the pollinator's body in *Galanthus nivalis* , but is not observed in *Ficaria verna* (Prokop et al. 2023). This supports the pollinator attraction hypothesis, which posits that specific floral orientations enhance floral detectability and visitation.

Floral orientation can further function as a selective filter, a mechanism crucial because not all flower visitors are effective pollinators. Ineffective pollinators, such as “pollen thieves” or “nectar robbers,” may fail to contact reproductive structures, consume floral rewards without transferring pollen grains, or even damage floral tissues due to mismatches in body size, morphology, or behavior (Fenster et al. 2004; Ye et al. 2017; Mackin et al. 2021). These interactions can negatively impact plant reproduction (Fenster et al. 2004; Mackin et al. 2021). Specific orientations can preferentially attract effective pollinators while deterring inefficient visitors (Wang, Xiao, et al. 2014; LoPresti et al. 2020), thereby providing support for the pollinator filtering hypothesis.

Concurrently, abiotic factors represent a significant selective force shaping the evolution of floral orientation (Hodges et al. 2002; Creux et al. 2021; Shibata et al. 2025). Downward corollas may serve a protective function against environmental factors such as rainfall and solar radiation, which are known to compromise pollen viability and stigma receptivity (Huang et al. 2002; Mao and Huang 2009; Wang et al. 2010). Previous studies have emphasized the role of floral orientation in mitigating rainwater‐induced damage, particularly in reducing pollen grain amount (Sun et al. 2018) and disrupting pollen viability in anthers (Huang et al. 2002; Nakata et al. 2022), and preventing nectar dilution of flowers (Aizen 2003); in contrast, direct experimental evidence regarding its protective effect against solar radiation remains limited (except Wang et al. 2010; Sun et al. 2018). Beyond these direct protective functions, the integrated effects of floral orientation on plant reproductive success through its interactions with both biotic and abiotic factors remain inadequately explored. Few studies have tracked the subsequent effects on plant reproductive success mediated by orientation changes (Sun and Yao 2013; Wang, Tie, et al. 2014). This knowledge gap limits our understanding of how floral orientation ultimately shapes plant reproductive success. Consequently, the comparative studies that quantitatively link floral orientation to comprehensive reproductive fitness still require further empirical evidence, particularly through systematic investigations of environmental adaptation in economically important plants.

*Polygonatum* Mill. (Asparagaceae) plants are widely distributed across the Northern Hemisphere, and the rhizomes of certain species within this genus are valued for their medicinal properties (Shi et al. 2023). Research on this genus primarily concentrates on chemical components and pharmacological effects (Mu et al. 2021; Lin et al. 2024; Liu et al. 2025), species classification and phylogenetic analysis (Hu et al. 2022; Xia et al. 2022), and the development of cultivation techniques (Zhao et al. 2025). Though reproductive ecology has been described for the genus (Liu et al. 2017; Li et al. 2021; Tang et al. 2025), the studies on pollinator interactions and floral functional traits remain limited. To our knowledge, no work has examined the functional ecology of downward tubular corolla orientation in *Polygonatum*. This study employs a comprehensive experimental approach to investigate the adaptive significance of floral orientation in *P. cyrtonema*. We conducted a manipulative experiment comparing inflorescences with manipulated upward (Up) flowers against those natural downward flowers (Control) to investigate the effects of floral orientation on pollination and plant reproductive success. We further evaluated the protective role of floral orientation by assessing pollen availability and stigma receptivity under varying conditions of sunlight and rainwater exposure. Our research addresses three questions as follows: (1) How does floral orientation affect pollinator assemblage, visitation rate, and pollen transfer efficiency? (2) How do sunlight exposure and rainwater affect pollen viability and stigma receptivity? (3) How does floral orientation influence reproductive fitness?

## 2 Materials and Methods

### 2.1 Study Species and Site

**FIGURE 1:**
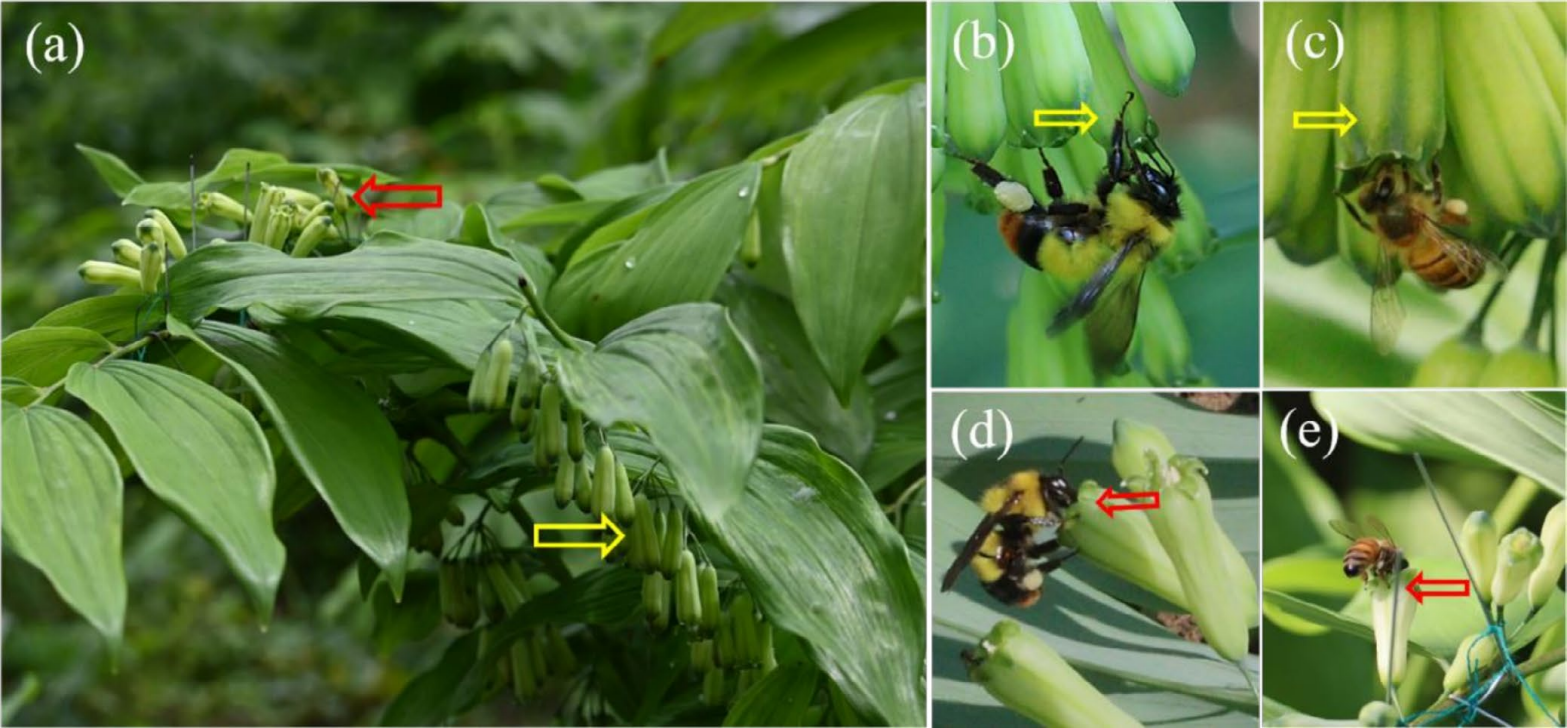
Floral orientation and pollinator visits in *Polygonatum cyrtonema*. (a) Downward (Control) and upward (Up) inflorescences; Bumble bee (b) and honey bee (c) on naturally downward flowers; Bumble bee (d) and honey bee (e) on manipulated upward flowers. Yellow and red arrows indicate Control and Up flowers, respectively. Photos were taken by Xiang‐xiang Ge and Ju Tang.

*Polygonatum cyrtonema* Hua. is a perennial herb and an official medicinal species recorded in the *Pharmacopoeia of the People's Republic of China* (Chinese Pharmacopoeia Commission 2020). It is naturally distributed across shaded environments, including forests, thickets, and slopes throughout southern and eastern China (Zhang et al. 2025). This plant is widely utilized in traditional Chinese medicine due to its rhizomes, which are rich in secondary metabolites such as triterpenoid saponins, flavonoids, and other nutritional components (Si and Zhu 2021; Shi et al. 2023; Liu et al. 2025). The plant blooms in spring and exhibits both sexual reproduction by seeds and asexual reproduction through the vegetative growth of the rhizomes. Individual plants produce multiple simple umbels, which are arranged in a racemose manner along the stem. The flower features a green perianth consisting of six tepals fused into a downward tubular corolla (Figure 1a shows the umbels and the tubular flowers). The androecium comprises six stamens with yellow anthers and the filaments are adnate to the corolla tube at their base. The gynoecium consists of three syncarpous carpels forming a superior ovary, topped by a trilobed stigma at maturity. A long style connects the stigma to the ovary, where each carpel contains approximately eight ovules. Nectar is secreted from nectaries located at the ovary base.

The field experiments were conducted in a natural population of *P. cyrtonema* located in Qingyang County, Anhui Province, China (30°63′ N, 117°84′ E, 440 m above sea level). The population consisted of more than 1000 flowering *P. cyrtonema* individuals, and the flowering period began in early May and lasted to early June. The flowers were frequently visited by bumble bees ( *Bombus trifasciatus* Smith) and honey bees ( *Apis mellifera* Linnaeus), with bumble bees being identified as the primary effective pollinators (Tang et al. 2025). The umbels at the stem apex of *P. cyrtonema* are later‐flowering (Figure 1a). To avoid potential effects of positional and phenological differences, flowers from the middle and lower parts of the stem were primarily selected for our experiments. Our field study was conducted from May 6 to 16 in 2024 and from May 10 to 22 in 2025, encompassing the flowering peak period at the study site.

### 2.2 Floral Orientation Manipulation Experiments

We employed two types of inflorescences with different floral orientations (Figure 1): (i) unmanipulated inflorescences with natural downward flowers (Control); (ii) manipulated inflorescences with artificially turned upward flowers (Up). The floral orientation was altered by gently bending the pedicel and securing the peduncle to the stalk with thin iron wire. To avoid damaging the floral buds, all manipulations were performed carefully to minimize plant disturbance. For both treatments (Up and Control), 15–20 individual plants were used for each floral orientation manipulation. On each plant, umbel inflorescences (totally 10–30 flowers per plant) at the bud stage were assigned to one orientation treatment.

### 2.3 Effects of Flower Orientation on Floral Longevity

To compare the floral longevity between natural (Control) and manipulated (Up) flowers, we randomly selected and marked two flower buds on each of 10 plants per treatment, that is, 20 flower buds per treatment in total (Up, *N* = 20 and Control, *N* = 20). Floral longevity was recorded as the number of days from anthesis until corolla wilting and abscission.

### 2.4 Effects of Flower Orientation on Pollinator Visitation

To evaluate whether floral visitors exhibit a preference between natural downward (Control) and artificially upward (Up) flowers, we compared visitation rate and foraging behavior between two orientation treatments. To ensure equivalent floral display, we selected 5–10 plant individuals and controlled the total flower count at the inflorescence level to ensure the number of open flowers of both treatments was similar during observation periods. Insects were considered pollinators only if they were observed transferring pollen grains of *P. cyrtonema* between flowers. Field observations of pollinators were conducted in three spatially independent plots, with a minimum distance of 5 m between any two plots. Each plot contained 20–50 flowering individuals. Observations were conducted on sunny and windless days between 8:30 and 17:30 when insects were active. Each plot was observed for at least 5 h in total. Two observers worked, and each observer was responsible for simultaneous observations of both the Up and Control flowers. The observers were trained well, demonstrating high consistency in identifying pollinators and behaviors before experiments. Pollinator activity was recorded in 30‐min intervals. During each interval, we recorded the insect species, number of visits per floral orientation, foraging behavior, time spent per flower (in seconds), and movements between flowers of different orientations. After pollinator observations, we counted the total number of Up and Control flowers at each plot to calculate the visitation rate (number of visits per flower per hour). To ensure accurate visitors' identification, approximately three individuals of each insect species observed foraging were captured upon leaving the observation plot for later identification.

### 2.5 Effects of Flower Orientation on Pollen Transfer

To compare pollen transfer efficiency between natural downward (Control) and artificially upward (Up) flowers, we examined pollen removal and pollen deposition per visit in 2024 and 2025. More than 50 flower buds from each orientation were enclosed in mesh bags to exclude pollinators until anthesis. Pollen removal and deposition per visit were measured specifically for bumble bees and honey bees, as visits by other visitors, such as syrphid flies, were too low to provide statistically meaningful data.

To estimate pollen removal per visit in both Control and Up flowers, the number of pollen grains was quantified by collecting flowers of each orientation after a single pollinator visit and preserving them separately in 75% ethanol. The number of pollen grains remaining in each flower was subsequently counted. In addition, 30 unopened flower buds were randomly collected to determine the average pollen production per flower. Pollen grains were released from the anthers using a homogenizer (FastPreP‐24, MP Biomedicals) operated for 14 s to rupture the anther sacs without damaging the pollen grains. The suspension was vortexed (Vortex‐2; Shanghai Huxi Industrial Co. Ltd.) to ensure homogeneity and then diluted to a final volume of 2 mL. From each sample, three 10 μL droplets were aspirated from the middle layer of the suspension and transferred to a glass slide. The number of pollen grains in each droplet (a₁, a₂, a₃) was counted under a microscope (NLCD 500, Jiangnan Yongxin Optics Co. Ltd.). The total number of pollen grains was estimated using the formula: (a₁ + a₂ + a₃)/3 × 200. Pollen removal per visit was calculated by subtracting the mean number of pollen grains remaining per flower from the average pollen production per flower.

To quantify pollen deposition on stigmas per visit in both Control and Up flowers, all bagged flower buds were emasculated using forceps before anther dehiscence. After a single bee visit, stigmas were collected and stored in 0.2 mL centrifuge tubes containing 75% ethanol. Each stigma was softened in 8 mol/L NaOH solution for 1 h and gently pressed onto a slide. The number of pollen grains on each stigma was counted under a microscope. The total pollen deposition per visit was quantified by counting all pollen grains from the ethanol storage tube, the NaOH solution, and those remaining on the stigma itself.

### 2.6 Effects of Flower Orientation on Pollen Viability and Stigma Receptivity

Given that solar radiation and rainwater are two major abiotic factors affecting gamete viability, we conducted separate experiments on sunny and rainy days to compare their relative impact on pollen viability and stigma receptivity in *P. cyrtonema*. All flowers were selected on the second day after anthesis, as our preliminary data indicated that both pollen viability and stigma receptivity peak at this stage. For the solar radiation experiment, 10 flowers (Up, *N* = 5; Control, *N* = 5) were exposed on sunny days. To test the effects of rainwater, another 10 flowers (Up, *N* = 5; Control, *N* = 5) were exposed during rainy days. Anthers were then collected from these flowers at 2‐h intervals between 08:00 and 18:00 (six time points daily). At each interval, pollen grains were sampled from one of the six anthers per flower to investigate the pollen viability (*N* = 5 per orientation per time point). Stigmas (Up, *N* = 5; Control, *N* = 5) were collected 24 h after the start of the treatment to assess the stigma receptivity. To evaluate the effect of rainwater exposure on pollen loss, we selected 10 newly opened flowers and artificially oriented them upward (Up, *N* = 10) during rainfall. Another 10 flowers in their natural orientation (Control, *N* = 10) that had opened on the same day were marked for comparison. After 10 h of natural rainfall (total precipitation: 12.6 mm), all floral samples were collected. The anthers were carefully excised and preserved in 75% ethanol. The remaining pollen grains were subsequently quantified under a microscope in the laboratory.

To assess pollen viability of two floral orientations (Control vs. Up) under both sunlight and rainwater exposures, pollen grains were collected from dehisced anthers of both groups. Anthers were gently tapped with tweezers to release pollen grains onto a glass slide with a 10 μL droplet of 0.1% MTT (3‐(4,5‐dimethyl‐2‐thiazolyl)‐2,5‐diphenyl‐2‐H‐tetrazolium bromide) solution. After 20 min of staining, the samples were observed under a microscope, and each droplet contained no less than 100 pollen grains. The numbers of darkly stained (viable) and total pollen grains were recorded. Pollen viability was calculated as the percentage of darkly stained grains.

To assess stigma receptivity of two floral orientations under both sunlight and rainwater exposures, all collected stigmas were carefully excised and placed on slides, then treated with one drop of 3% H_2_O_2_ solution. The stigma receptivity was assessed by counting the number of bubbles produced around the stigma within 1 min. Bubble formation indicates peroxidase activity, which serves as an indicator of stigma receptivity.

### 2.7 Effects of Flower Orientation on Reproductive Fitness

To evaluate the effects of floral orientation on reproductive fitness, we compared fruit and seed production between naturally downward (Control) and artificially upward (Up) flowers. Twenty individual plants were randomly selected and assigned to Control (*N* = 10) or Up (*N* = 10) treatments, with an average of 21.2 ± 1.7 flowers per plant in 2025 and 23.4 ± 2.3 flowers per plant in 2024. Following 3 days of exposure to natural pollinators after anthesis, stigmas were collected from those flowers of both treatments. Pollen grains deposited on the stigmas were quantified under a microscope. The total number of treated flowers per plant was recorded for subsequent calculations. Approximately 3 months later, fruits were harvested to assess fruit set and seed set per treatment. We also measured fruit and seed quality traits, including fruit size (suture and polar diameters), fruit fresh weight, seed diameter, and seed number per fruit for both orientation treatments.

### 2.8 Data Analysis

Statistical analyses were conducted using generalized linear models (GLMs) and Mann–Whitney *U*‐test (M–W *U*‐test) in IBM SPSS Statistics (27.0; IBM Corp., NY, USA). Treatment differences and effects were considered statistically significant at *p* < 0.05. Data visualization was performed using OriginPro (9.5, OriginLab Corp., MA, USA), and final figures were compiled in Adobe Photoshop CS6 (13.0; Adobe Inc., CA, USA).

To analyze the effects of flower orientation (Control vs. Up) on pollinator visitation rate, we used the M–W *U*‐test. We employed GLMs with a normal distribution and an identity link function to analyze the effects on visiting time per flower and key reproductive fitness components. Visiting time per flower, fruit fresh weight, fruit size (suture and polar diameters) and seed diameter were the dependent variables. Data for visiting time per flower were log‐transformed to meet the assumption of normality for model residuals. The normality of residuals for all GLMs was confirmed using the Shapiro–Wilk test (*p* > 0.05). Pollen viability under varying weather conditions was analyzed using repeated‐measures ANOVA within a general linear model to assess time, orientation, and weather effects. Since the number of bubbles on the stigma, the number of pollen grains (including pollen removal and deposition per visit, and pollen load on stigmas) and seed number per fruit represent count data, these variables were analyzed using GLMs with Poisson distribution and log‐linear link function. Floral longevity was also used GLMs with a Poisson distribution and log‐linear link function. Fruit set and seed set were analyzed using GLMs with a binomial distribution and logit link function. For fruit set, the model specified the number of developed fruits as the events and the total treated flower number per plant as the trials. Similarly, for seed set, the model used the number of developed seeds as the events and the total ovule number as the trials. Flower orientation (Control vs. Up) as the fixed factor in all models.

## 3 Results

### 3.1 Effects of Flower Orientation on Floral Longevity

Individual floral longevity ranged from 2 to 5 days. Naturally downward (Control) flowers exhibited significantly longer longevity (3.79 ± 0.16 days, *N* = 19) than those artificially oriented upward (Up) flowers (2.65 ± 0.13 days, *N* = 20; Wald χ^2^ = 28.626, *p* < 0.001).

### 3.2 Effects of Flower Orientation on Pollinator Visitation

**FIGURE 2:**
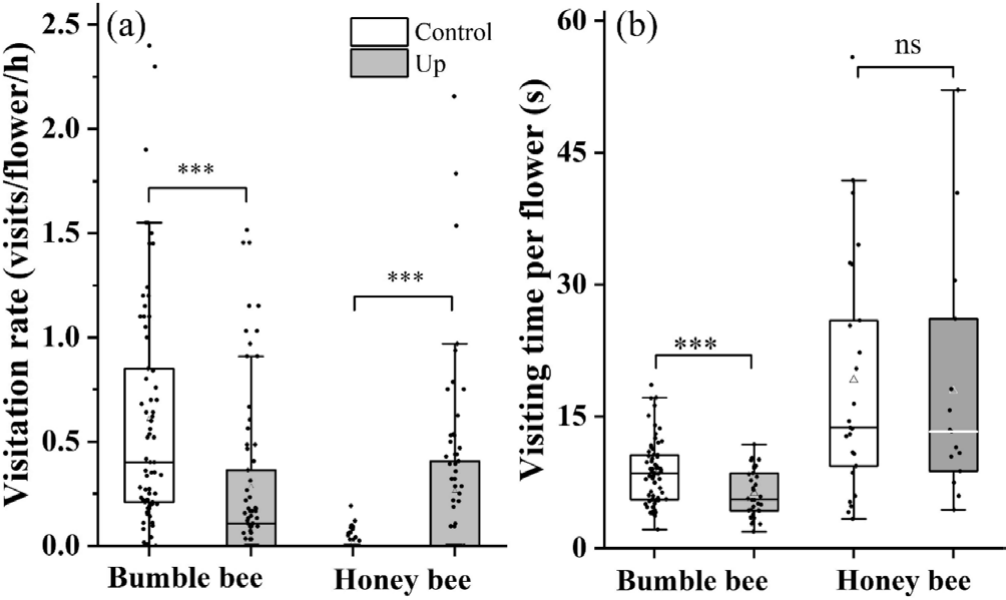
Visitation rate (a) and visiting time per flower (b) by bumble bees and honey bees on downward (Control, open boxs) and upward (Up, solid gray boxs) flowers of *Polygonatum cyrtonema*. All panels display box plots based on raw data (solid dots), indicating the mean (triangles), median (horizontal lines), interquartile range (the upper and lower edges of the box), 1.5 times the interquartile range (whiskers) and outliers. ***p < 0.001, and ns indicates no significant differences between flower orientations.

Field observations conducted over 2024 and 2025 showed that bumble bees ( *Bombus trifasciatus* ) and honey bees ( *Apis mellifera* ) were the pollinators of *P. cyrtonema*. Syrphid flies were infrequently observed visiting flowers and rarely carried pollen. Bumble bees were the predominant effective pollinator group, while honey bees were occasionally recorded visiting flowers in 2025. Based on a total of 69 observation periods (34.5 h), bumble bees consistently visited Control flowers more frequently than Up flowers. Both bee species foraged for nectar by inserting their bodies into the floral tube, leading to pollen adhesion on their heads and thoraxes (Figure 1b–e). These pollen grains were deposited on stigmas during subsequent floral visits or collected into the corbiculae on their hind legs (Figure 1b–e). In 2024 and 2025, the visitation rate of Control flowers by bumble bees was significantly higher than that to Up flowers (*U* = 1169.500, *p* < 0.001; Figure 2a). In contrast, honey bees exhibited the opposite trend, visiting Up flowers at a significantly higher rate than Control flowers (*U* = 1583.500, *p* < 0.001; Figure 2a). Upward flowers attracted more visits of syrphid flies with a higher visitation rate (0.015 ± 0.006 visits/flower/h) than downward flowers (0.005 ± 0.003 visits/flower/h).

The average visiting time per flower by bumble bees was significantly longer for Control flowers (8.58 ± 0.44 s, *N* = 67) than for Up flowers (6.23 ± 0.46 s, *N* = 34; Wald χ^2^ = 12.130, *p* < 0.001). While there was no significant difference in visit duration between Control (19.12 ± 2.76 s, *N* = 25) and Up (17.92 ± 3.53 s, *N* = 15) for honey bees (Wald χ^2^ = 0.019, *p* = 0.890 > 0.05; Figure 2b).

### 3.3 Effects of Flower Orientation on Pollen Transfer

**FIGURE 3:**
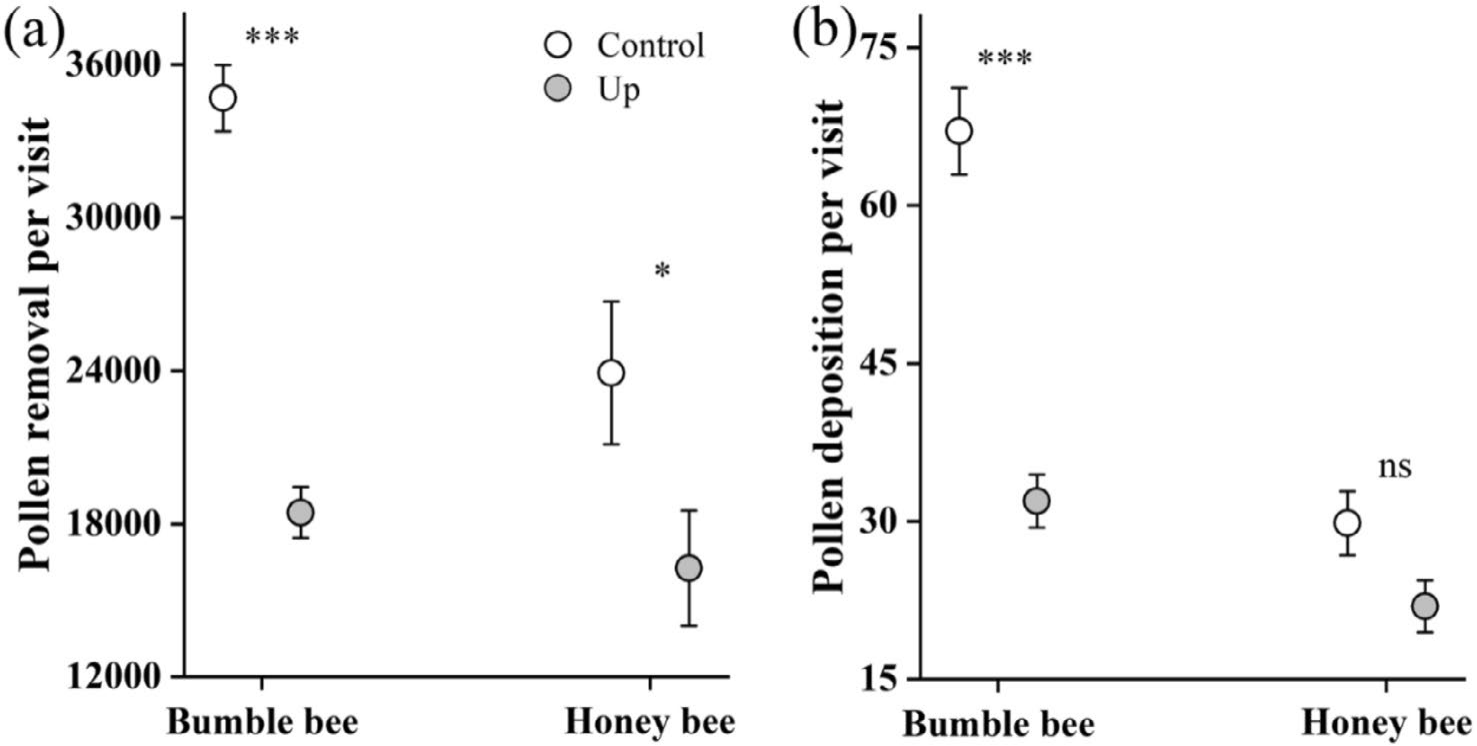
Pollen removal (a) and deposition (b) per visit by bumble bees and honey bees on downward (Control, open circles) and upward (Up, solid gray circles) flowers of *Polygonatum cyrtonema*. Bars show standard errors. ***p < 0.001, *p < 0.05, and ns indicates no significant differences.

The mean number of pollen grains per flower was 94,313.3 ± 1774.2 (*N* = 30). Pollen removal per visit by bumble bees was significantly higher than that by honey bees on natural flowers (Wald χ^2^ = 13.175, *p* < 0.001). Bumble bees removed significantly more pollen grains per visit on Control flowers (34,694.3 ± 1296.6, *N* = 48) than on Up flowers (18,445.5 ± 997.9, *N* = 38; Wald χ^2^ = 83.498, *p* < 0.001; Figure 3a). Similarly, honey bees removed more pollen grains on Control flowers (23,921.3 ± 2793.1, *N* = 30) than on Up flowers (16,259.4 ± 2256.9, *N* = 28; Wald χ^2^ = 4.472, *p* = 0.034; Figure 3a).

Bumble bees deposited significantly more pollen grains per visit than honey bees (Wald χ^2^ = 44.223, *p* < 0.001). When visiting Control flowers, bumble bees deposited over twice as many pollen grains (67.1 ± 4.1, *N* = 87) compared to Up flowers (31.9 ± 2.5, *N* = 51; Wald χ^2^ = 48.814, *p* < 0.001; Figure 3b). In contrast, honey bees showed a marginally significant difference in pollen deposition per visit between downward (29.8 ± 3.0, *N* = 45) and upward flowers (21.9 ± 2.5, *N* = 33; Wald χ^2^ = 3.808, *p* = 0.051; Figure 3b).

### 3.4 Effects of Flower Orientation on Pollen Viability and Stigma Receptivity

**FIGURE 4:**
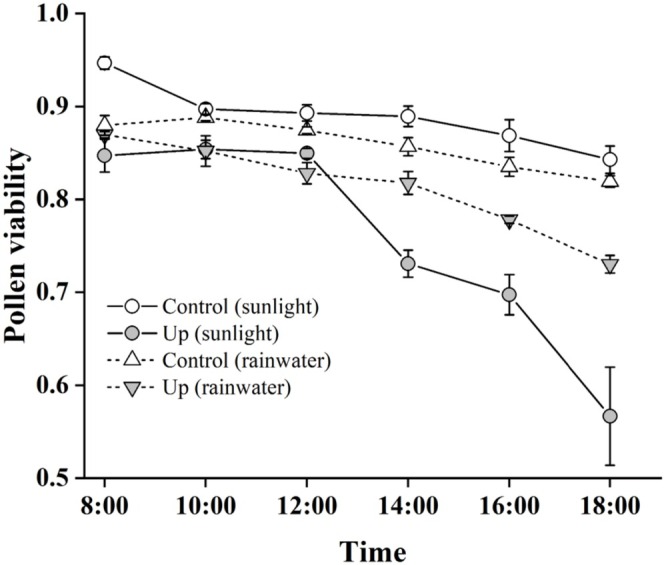
Temporal changes in pollen viability of *Polygonatum cyrtonema* under sunlight (circles, solid lines) and rainwater (triangles, dash lines) exposure over a 10‐h period (08:00–18:00) in 2025. Viability was assessed at 2‐h intervals using 0.1% MTT solution. Bars show standard errors.

Pollen viability in *P. cyrtonema* was significantly affected by sampling time, floral orientation and weather conditions (sunlight vs. rainwater exposure; Table 1; Figure 4). Overall, downward flowers maintained higher pollen viability than upward flowers throughout the experimental period. A general decline in pollen viability was observed with increasing exposure time under all conditions (Figure 4). Under sunlight, the pollen viability of downward flower was 0.95 ± 0.01 at 08:00 and gradually declined to 0.84 ± 0.01 by 18:00. However, this decline was significantly more pronounced in Up flowers with prolonged exposure time (*p* < 0.001; Figure 4). Notably, after 6 h of exposure (14:00), pollen viability decreased more rapidly under sunlight than under rainwater (Figure 4), indicating that solar radiation causes greater damage to pollen grains than rainfall. The significant two‐way interactions were detected between time and orientation (*p* < 0.001) and between time and weather condition (*p* = 0.014), demonstrating that the rate of pollen viability decline differed between floral orientations and weather treatments (Table 1). A significant three‐way interaction (time × orientation × weather condition, *p* = 0.012) further indicated that the temporal dynamics of pollen viability depended on the combined effects of orientation and weather conditions (Table 1). Additionally, Up flowers experienced greater pollen loss from rainwater washing. After 10 h of rainfall, Control flowers retained significantly more pollen grains (83,380 ± 2281.9, *N* = 10) than Up flowers (57,546.7 ± 2976.5, *N* = 10; Wald χ^2^ = 44.730, *p* < 0.001).

**TABLE 1:** Results of the general linear model analyzing the effects of time, floral orientation, and weather condition on pollen viability in *Polygonatum cyrtonema*.

Source of variation	df	*F*	*p*
Within‐subjects effects			
Time	2.044, 32.700	47.424	< 0.001***
Time × Orientation	2.044, 32.700	11.608	< 0.001***
Time × Condition	2.044, 32.700	4.808	0.014*
Time × Orientation × Condition	2.044, 32.700	5.068	0.012*
Between‐subjects effects			
Orientation	1, 16	343.81	< 0.001***
Condition	1, 16	6.41	0.022*
Orientation × Condition	1, 16	79.776	< 0.001***

*Note:* The Greenhouse–Geisser correction was applied (ε = 0.803). Degrees of freedom were adjusted accordingly following a significant Mauchly's test of sphericity (*p* < 0.05). ***p < 0.001, *p < 0.05.

Stigma receptivity was significantly influenced by both floral orientation and weather conditions. After 24 h of sunlight exposure, Control flowers produced substantially more bubbles on stigmas (20.0 ± 2.3, *N* = 5) than Up flowers (6.6 ± 0.9, *N* = 5; Wald χ^2^ = 31.440, *p* < 0.001). Similarly, after 24 h of rainfall, Control flowers showed higher bubble counts (18.0 ± 2.2, *N* = 5) compared to Up flowers (13.0 ± 1.2, *N* = 5; Wald χ^2^ = 4.105, *p* = 0.043).

### 3.5 Effects of Flower Orientation on Reproductive Fitness

The reproductive fitness of downward flowers was overall higher than that of upward flowers. Up flowers received significantly fewer pollen grains (27.4 ± 3.0, *N* = 7) than Control flowers (104.2 ± 9.7, *N* = 30; Wald χ^2^ = 14.787, *p* < 0.001). Fruit fresh weight and size (suture and polar diameters) were significantly greater in downward flowers (all *p* < 0.05; Table 2). Downward flowers also produced higher seed set, more seeds per fruit, and larger seeds than upward flowers (all *p* < 0.05; Table 2). Although fruit set did not differ significantly between treatments with the current sample size (*N* = 10 plants per group; *p* > 0.05; Table 2), a consistent trend of lower fruit set in upward plants was observed across both 2 years.

**TABLE 2:** Reproductive fitness components (mean ± SE) of *Polygonatum cyrtonema* under natural downward (Control) and manipulated upward (Up) floral orientations.

	Fitness components	Control	Up	Wald χ^2^	*p*
Fruit	Fruit set per plant (2025)	0.54 ± 0.09 (10)	0.42 ± 0.09 (10)	2.261	0.133
	Fruit set per plant (2024)	0.22 ± 0.06 (11)	0.09 ± 0.04 (10)	1.513	0.219
	Fresh weight per fruit (g)	**0.95 ± 0.07** (30)	0.69 ± 0.03 (30)	11.521	< 0.001***
	Fruit suture diameter (mm)	**11.59 ± 0.29** (30)	10.60 ± 0.17 (30)	7.955	0.005**
	Fruit polar diameter (mm)	**11.04 ± 0.25** (30)	9.88 ± 0.19 (30)	13.280	< 0.001***
Seed	Seed set	**0.40 ± 0.03** (30)	0.31 ± 0.02 (30)	6.182	0.013*
	Seed number per fruit	**7.2 ± 0.5** (30)	5.6 ± 0.3 (30)	6.397	0.011*
	Seed diameter (mm)	**4.69 ± 0.04** (215)	4.2 ± 0.04 (168)	71.560	< 0.001***

*Note:* Bold values denote statistically significant differences between two orientations. Sample sizes are shown in parentheses. ***p < 0.001, **p < 0.01, *p < 0.05.

## 4 Discussion

In this study, we conducted a manipulative experiment comparing inflorescences with artificially oriented Up flowers against natural downward (Control) flowers to investigate the effects of floral orientation on pollination processes, protection against sunlight and rainwater, and overall reproductive success in *P. cyrtonema*. Our results demonstrate that the downward floral orientation in *P. cyrtonema* functions as an adaptive trait that enhances reproductive success through protecting pollen grains and stigmas from environmental stress, increasing attraction to effective bumble bee pollinators, reducing visits from less efficient honey bees and syrphid flies, and improving the efficiency of pollen transfer. Although fruit set did not differ significantly between Up and Control orientations, Control flowers produced larger and higher quality fruits and seeds, as well as significantly higher seed set and a greater number of swollen seeds per fruit compared to Up flowers. These findings demonstrate that the downward tubular corolla in *P. cyrtonema* improves reproductive fitness, thus underscoring floral orientation as a key adaptive trait.

### 4.1 Effects of Floral Orientation on Pollinator Behavior and Pollen Transfer

In agreement with other studies, our results support the idea that floral orientation influences pollinator attraction, particularly in structuring pollinator assemblages and visitation frequency (Fulton and Hodges 1999; Ushimaru and Hyodo 2005; Wang, Tie, et al. 2014; Wang, Xiao, et al. 2014). For example, a study on upward *Aquilegia pubescens* showed that altering floral orientation reduced visitation by its effective pollinators, hawk moths (Fulton and Hodges 1999), whereas naturally downward flowers of *Geranium refractum* effectively excluded inefficient visitors while attracting more effective pollinators (Wang, Xiao, et al. 2014). The bumble bees, which are the primary effective pollinators of *P. cyrtonema* (Tang et al. 2025), exhibited a strong preference for naturally oriented downward flowers. By contrast, honey bees and other syrphid flies were less effective at pollen removal and deposition, yet they exhibited increased visits to Up flowers. This pattern aligns with the behavior of naive pollinators documented in the snowdrop *Galanthus nivalis* , where a lack of prior experience can lead to increased investigation of novel floral orientation displays (Prokop et al. 2020). The observed shift in honey bee visitation toward manipulated flowers may therefore reflect their relatively lower prior specialization on *P. cyrtonema* compared to bumble bees, which are morphologically better adapted to floral architecture and are more effective pollinators (Tang et al. 2025). Collectively, these results indicate that the downward floral orientation functions as a selective filter, preferentially attracting efficient bumble bees while excluding or reducing visitation by less effective visitors.

Moreover, bumble bees spent more time on naturally oriented flowers and transferred significantly more pollen grains per visit, indicating that the downward orientation not only attracts the effective pollinators but also facilitates pollen transfer efficiency, thereby providing greater opportunities to promote reproductive success (Fenster et al. 2004; Prokop et al. 2023). Consequently, the reduced visitation rate and shorter duration on upward flowers likely limited both male fitness through diminished pollen export and female fitness through lower pollen receipt. This is consistent with the findings that altering floral orientation can disrupt pollinator landing behavior and handling efficiency (Ushimaru et al. 2009; Wang, Tie, et al. 2014; Wang, Xiao, et al. 2014; Prokop et al. 2023). In summary, the natural downward orientation confers a reproductive advantage through enhanced pollinator attraction and pollen transfer. Our results support both the pollinator attraction and filtering hypotheses, demonstrating floral orientation to be a significant adaptive strategy in *P. cyrtonema*.

### 4.2 The Protective Effects of Downward Flowers Against Solar Radiation and Rainwater

Our results are consistent with the idea that the downward floral orientation of plants often serves as a protective mechanism by sheltering reproductive structures from exposure to sunlight and rainwater, reducing damage to pollen grains and stigmas (Huang et al. 2002; Wang et al. 2010; Nakata et al. 2022). For example, the pendulous flowers of *Anisodus luridus* protect pollen grains from both solar radiation and rainwater, enhancing male fitness (Wang et al. 2010). Similarly, the petals of *Pulsatilla cernua* function like an umbrella to reduce rain damage (Huang et al. 2002). Our study in *P. cyrtonema* also provides strong support for the protective hypothesis of floral orientation in protecting pollen grains quantity, pollen viability, and stigma receptivity. Both pollen grains quantity and quality were significantly decreased in Up flowers. Downward flowers maintained higher pollen viability and stigma receptivity compared to Up flowers. Furthermore, Up flowers experienced greater pollen grains loss with less pollen grains retained in anthers after 10 h of natural rainfall. By shielding pollen from intense solar radiation and reducing pollen loss from rainwater washout, the downward tubular corolla of *P. cyrtonema* flowers thereby helps to protect the male and female gametes to maintain their function, enhancing the probability of successful pollination during subsequent visits. Moreover, the maintenance of pollen viability and stigma receptivity prolongs the functional window for pollinator visits, thus enhancing reproductive opportunity. Upward flowers of *P. cyrtonema* withered more rapidly than natural downward flowers. This finding is consistent with observations in *Prunus mume* , in which downward flowers also displayed significantly longer floral longevity than upward flowers (Ge et al. 2025). The accelerated senescence in upward flowers is likely due to the direct exposure of reproductive organs to environmental stressors such as solar radiation and rainwater. The extended floral longevity, coupled with greater pollen grain and stigma performance in downward flowers may ultimately improve reproductive success.

A downward orientation can also protect floral nectar from dilution by rainfall, thereby maintaining its attractiveness and reward for pollinators (Aizen 2003). While our study focused primarily on how orientation affects pollen transfer and reproductive success via pollen viability and stigma receptivity, future investigations incorporating nectar dynamics would provide a more integrated understanding of floral adaptation. Our study highlights the critical role of floral orientation in shielding reproductive tissues not only from rainwater but also from solar radiation damage. The viability of pollen grains and stigmas exposed to solar radiation decreased greatly than those exposed to rainwater, revealing that solar radiation exerts a greater negative impact on both pollen viability and stigma receptivity. Numerous studies have confirmed that pollen grains are sensitive to UV‐B radiation by reducing viability and/or disrupting germination (Feng et al. 2000; Wang et al. 2010; Cun et al. 2024). The flowering of *P. cyrtonema* in early summer coincides with the seasonal increase in solar radiation. Moreover, direct exposure to sunlight could increase the temperature and thus induce the damage to the pollen grains and stigmas. Downward flowers significantly reduce solar radiation damage in *P. cyrtonema*.

In *P. cyrtonema*, some pollen grains were observed to burst when exposed to rainwater. This result is consistent with several other terrestrial species (Huang et al. 2002; Mao and Huang 2009). Pollen germination in water is generally poor, with the resulting pollen tubes being short (Vasil 1960; Johri and Vasil 1961). Interestingly, the proportion of germinating pollen grains initially exceeded that of bursting pollen grains, but the proportion of bursting pollen grains increased over time in our study. Pollen viability in *P. cyrtonema* was high (> 70%) even after rainwater exposure after 10 h in natural flowers. This suggests that pollen grains in *P. cyrtonema* possess traits enabling not only a degree of water tolerance but also controlled hydration. A well‐structured exine and the presence of hydrophilic proteins or polysaccharides in the pollen coat could moderate water absorption (Nepi et al. 2001; Edlund et al. 2004). This mechanism remains to be fully confirmed for future research through the architecture and biochemistry studies on pollen grains in *P. cyrtonema*.

### 4.3 Effects of Floral Orientation on Reproductive Success

Floral orientation as a key adaptive trait that significantly influences plant reproductive success has been demonstrated in multiple plant systems. In *Lilium duchartrei*, naturally downward flowers produced significantly higher seed set compared to flowers that had their direction changed (Sun and Yao 2013). Similarly, experimental manipulation of inflorescence orientation in *Corydalis sheareri* resulted in markedly reduced seed set (Wang, Tie, et al. 2014). These patterns were strongly supported in *P. cyrtonema*. Upward inflorescences consistently had lower fruit set in both years, together with reduced seed set, fewer seeds per fruit, and smaller seeds compared to downward inflorescences.

The downward orientation functions as an integrated trait that improves reproductive success (Fenster et al. 2004; Huang et al. 2002; Wang et al. 2010). First, the natural downward corolla provides essential protection against solar radiation and rainwater exposure (Huang et al. 2002; Wang et al. 2010), thereby maintaining pollen viability and stigma receptivity in *P. cyrtonema*. Second, this orientation facilitates pollen transfer efficiency, whereas artificial reorientation reduced visitation rate and pollen transfer. The substantially higher pollen deposition on stigmas of downward flowers compared to upward flowers reflects enhanced male fitness through more efficient pollen export and transfer.

In conclusion, our findings demonstrate that the naturally downward floral orientation in *P. cyrtonema* enhances reproductive fitness through both optimized pollination processes and abiotic protection. The downward orientation significantly improved pollen viability and stigma receptivity, increased visitation by effective pollinators, and ultimately led to higher seed set and seed quality, thereby improving both male and female reproductive fitness. This combination of advantages underscores the evolutionary significance of floral orientation in maximizing reproductive success under natural conditions.

## Author Contributions

**Ju Tang:** conceptualization (equal), data curation (equal), formal analysis (lead), funding acquisition (equal), methodology (equal), visualization (lead), writing – original draft (lead), writing – review and editing (equal). **Deng‐fei Li:** conceptualization (equal), methodology (equal), writing – review and editing (equal). **Xiang‐xiang Ge:** data curation (equal), investigation (equal), methodology (equal). **Yu‐jie Xu:** investigation (equal), methodology (equal). **Jian‐wen Shao:** conceptualization (equal), funding acquisition (equal), methodology (equal), writing – review and editing (equal).

## Funding

This study was supported by grants from the National Natural Science Foundation of China (32200184, 32470380) and the Anhui Provincial Natural Science Foundation (2208085QC70).

## Conflicts of Interest

The authors declare no conflicts of interest.

## Supporting information


**Data S1:** ece373221‐sup‐0001‐DataS1.xlsx.

## Data Availability

Data relevant to this paper that support the findings of this study are openly available in Supporting Information.

## References

[ece373221-bib-0001] Aizen, M. A. 2003. “Down‐Facing Flowers, Hummingbirds and Rain.” Taxon 52: 675–680. 10.2307/3647342.

[ece373221-bib-0002] Chinese Pharmacopoeia Commission . 2020. Pharmacopoeia of the People's Republic of China (Part I) (In Chinese). 2020th ed. China Pharmaceutical Science and Technology Press.

[ece373221-bib-0003] Creux, N. M. , R. I. Brown , A. G. Garner , et al. 2021. “Flower Orientation Influences Floral Temperature, Pollinator Visits and Plant Fitness.” New Phytologist 232: 868–879. 10.1111/nph.17627. 34318484

[ece373221-bib-0004] Cun, S. , C. Zhang , J. Chen , L. Qian , H. Sun , and B. Song . 2024. “Effects of UV‐B Radiation on Pollen Germination and Tube Growth: A Global Meta–Analysis.” Science of the Total Environment 915: 170097. 10.1016/j.scitotenv.2024.170097. 38224898

[ece373221-bib-0005] Edlund, A. F. , R. Swanson , and D. Preuss . 2004. “Pollen and Stigma Structure and Function: The Role of Diversity in Pollination.” Plant Cell 16, no. Suppl: S84–S97. 10.1105/tpc.015800. 15075396 PMC2643401

[ece373221-bib-0006] Feng, H. Y. , L. Z. An , L. L. Tan , Z. Hou , and X. Wang . 2000. “Effect of Enhanced Ultraviolet–B Radiation on Pollen Germination and Tube Growth of 19 Taxa In Vitro.” Environmental and Experimental Botany 43: 45–53. 10.1016/S0098-8472(99)00042-8.

[ece373221-bib-0007] Fenster, C. B. , W. S. Armbruster , and M. R. Dudash . 2009. “Specialization of Flowers: Is Floral Orientation an Overlooked First Step?” New Phytologist 183: 502–506. 10.1111/j.1469-8137.2009.02852.x. 19422542

[ece373221-bib-0008] Fenster, C. B. , W. S. Armbruster , P. Wilson , M. R. Dudash , and J. D. Thomson . 2004. “Pollination Syndromes and Floral Specialization.” Annual Review of Ecology, Evolution, and Systematics 35: 375–403. 10.1146/annurev.ecolsys.34.011802.132347.

[ece373221-bib-0009] Fulton, M. , and S. A. Hodges . 1999. “Floral Isolation Between *Aquilegia formosa* and *A. pubescens* .” Proceedings of the Royal Society B: Biological Sciences 266: 2247–2252. 10.1098/rspb.1999.0915.

[ece373221-bib-0010] Ge, X. X. , Y. J. Xu , and J. Tang . 2025. “Effect of Flower Orientation Variation on Pollinator Foraging Behavior and Pollination Efficiency in *Prunus mume* f. *purpurea* .” Biodiversity Science 33, no. 9: 25221. 10.17520/biods.2025221.

[ece373221-bib-0011] Hodges, S. A. , J. B. Whittall , M. Fulton , and J. Y. Yang . 2002. “Genetics of Floral Traits Influencing Reproductive Isolation Between *Aquilegia formosa* and *Aquilegia pubescens* .” American Naturalist 159: S51–S60. 10.1086/338372 18707369

[ece373221-bib-0012] Hu, Y. , Y. Liu , M. Ali , et al. 2022. “ *Polygonatum praecox* (Asparagaceae), a New Species From Mid‐Eastern China Revealed by Morphological and Molecular Evidence.” PhytoKeys 211: 125–138. 10.3897/phytokeys.211.90456. 36760726 PMC9878575

[ece373221-bib-0013] Huang, S. Q. , Y. Takahashi , and A. Dafni . 2002. “Why Does the Flower Stalk of *Pulsatilla cernua* (Ranunculaceae) Bend During Anthesis?” American Journal of Botany 89: 1599–1603. 10.3732/ajb.89.10.1599. 21665586

[ece373221-bib-0014] Johri, B. M. , and I. K. Vasil . 1961. “Physiology of Pollen.” Botanical Review 27: 325–381. 10.1007/BF02860810.

[ece373221-bib-0015] Li, L. G. , Z. R. Zhang , Y. Shi , et al. 2021. “Investigation on Reproductive Characteristics of *Polygonatum cyrtonema* .” Zhongguo Zhong Yao Za Zhi 46: 1079–1083. 10.19540/j.cnki.cjcmm.20201223.101. 33787100

[ece373221-bib-0016] Lin, H. , W. Wang , M. Peng , et al. 2024. “Pharmacological Properties of *Polygonatum* and Its Active Ingredients for the Prevention and Treatment of Cardiovascular Diseases.” Chinese Medicine 19: 1. 10.1186/s13020-023-00871-0. 38163901 PMC10759625

[ece373221-bib-0017] Liu, C. , Y. Cao , Y. Zhao , S. Lu , and Q. Xia . 2025. “Protein Composition and Nutritional Evaluation of Three *Polygonatum* Mill Species: A Comparative Analysis.” Food Chemistry: X 27: 102390. 10.1016/j.fochx.2025.102390. 40206048 PMC11979906

[ece373221-bib-0018] Liu, J. , W. X. Wang , X. Zhu , et al. 2017. “Study on Floral Dynamics and Pollination of *Polygonatum cyrtonema* .” Seed 36: 41–45.

[ece373221-bib-0019] LoPresti, E. F. , J. Goidell , J. M. Mola , et al. 2020. “A Lever Action Hypothesis for Pendulous Hummingbird Flowers: Experimental Evidence From a Columbine.” Annals of Botany 125: 59–65. 31402377 10.1093/aob/mcz134 PMC6948206

[ece373221-bib-0020] Mackin, C. R. , D. Goulson , and M. C. Castellanos . 2021. “Novel Nectar Robbing Negatively Affects Reproduction in *Digitalis purpurea* .” Ecology and Evolution 11: 13455–13463. 10.1002/ece3.8068. 34646482 PMC8495828

[ece373221-bib-0021] Mao, Y. Y. , and S. Q. Huang . 2009. “Pollen Resistance to Water in 80 Angiosperm Species: Flower Structures Protect Rain‐Susceptible Pollen.” New Phytologist 183: 892–899. 10.1111/j.1469-8137.2009.02925.x. 19563452

[ece373221-bib-0022] Mu, C. , Y. Sheng , Q. Wang , A. Amin , X. Li , and Y. Xie . 2021. “Potential Compound From Herbal Food of *Rhizoma polygonati* for Treatment of COVID‐19 Analyzed by Network Pharmacology: Viral and Cancer Signaling Mechanisms.” Journal of Functional Foods 77: 104149. 10.1016/j.jff.2020.104149. 32837538 PMC7427583

[ece373221-bib-0023] Nakata, T. , I. Rin , Y. A. Yaida , and A. Ushimaru . 2022. “Horizontal Orientation Facilitates Pollen Transfer and Rain Damage Avoidance in Actinomorphic Flowers of *Platycodon grandiflorus* .” Plant Biology 24: 798–805. 10.1111/plb.13414. 35289975

[ece373221-bib-0024] Nepi, M. , G. G. Franchi , and E. Padni . 2001. “Pollen Hydration Status at Dispersal: Cytophysiological Features and Strategies.” Protoplasma 216: 171–180. 10.1007/BF02673869. 11732185

[ece373221-bib-0025] Nevard, L. , and M. Vallejo‐Marín . 2022. “Floral Orientation Affects Outcross‐Pollen Deposition in Buzz‐Pollinated Flowers With Bilateral Symmetry.” American Journal of Botany 109: 1568–1578. 10.1002/ajb2.16078. 36193950 PMC9828177

[ece373221-bib-0026] Prokop, P. , Z. Ježová , M. Mešková , V. Vanerková , M. Zvaríková , and P. Fedor . 2023. “Flower Angle Favors Pollen Export Efficiency in the Snowdrop *Galanthus nivalis* (Linnaeus, 1753) but Not in the Lesser Celandine *Ficaria verna* (Huds, 1762).” Plant Signaling & Behavior 18: 2163065. 10.1080/15592324.2022.2163065. 36635990 PMC9851262

[ece373221-bib-0027] Prokop, P. , M. Zvaríková , Z. Ježová , et al. 2020. “Functional Significance of Flower Orientation and Green Marks on Tepals in the Snowdrop *Galanthus nivalis* (Linnaeus, 1753).” Plant Signaling & Behavior 15: 1807153. 32799622 10.1080/15592324.2020.1807153 PMC7588181

[ece373221-bib-0028] Shi, Y. , J. Liu , D. Si , et al. 2023. “Huangjing—From Medicine to Healthy Food and Diet.” Food Frontiers 4: 1068–1090. 10.1002/fft2.231.

[ece373221-bib-0029] Shibata, A. , G. Yumoto , H. Shimizu , M. N. Honjo , and H. Kudoh . 2025. “Flower Movement Induced by Weather‐Dependent Tropism Satisfies Attraction and Protection.” Nature Communications 16: 4132. 10.1038/s41467-025-59337-6. PMC12049521 40319049

[ece373221-bib-0030] Si, J. P. , and Y. X. Zhu . 2021. “ *Polygonati rhizoma*—A New High–Quality Crop With Great Potential and Not Occupying Farmland (In Chinese).” Science China. Life Sciences 51: 1477–1484. 10.1360/SSV-2020-0413. PMC8218280 34156598

[ece373221-bib-0031] Sun, K. , B. Q. Fan , Q. Z. Hou , et al. 2018. “The Adaptive Significances of Down Ward Orientation Flowers in Alpine Species *Clematis tangutica* .” Northwest Normal University Journal (Natural Science) 54: 55–60. 10.7606/j.issn.1000-4025.2016.11.2283.

[ece373221-bib-0032] Sun, S. G. , and C. Y. Yao . 2013. “Increased Seed Set in Down Slope‐Facing Flowers of *Lilium duchartrei* .” Journal of Systematics and Evolution 51: 405–412. 10.1111/jse.12002.

[ece373221-bib-0033] Tadey, M. , and M. A. Aizen . 2001. “Why Do Flowers of a Hummingbird‐Pollinated Mistletoe Face Down?” Functional Ecology 15: 782–790. 10.1046/j.0269-8463.2001.00580.x.

[ece373221-bib-0034] Tang, J. , X. X. Ge , Y. J. Xu , Y. Zhang , J. W. Shao , and X. H. Li . 2025. “A Comparison of Pollination Efficiency Between Wild Bumble Bees and Introduced Honey Bees on *Polygonatum cyrtonema* .” Biology 14: 276. 10.3390/biology14030276. 40136532 PMC11940143

[ece373221-bib-0035] Tong, Z. Y. , L. Y. Wu , H. H. Feng , et al. 2023. “New Calculations Indicate That 90% of Flowering Plant Species Are Animal–Pollinated.” National Science Review 10: nwad219. 10.1093/nsr/nwad219. 37743955 PMC10517183

[ece373221-bib-0036] Ushimaru, A. , I. Dohzono , Y. Takami , and F. Hyodo . 2009. “Flower Orientation Enhances Pollen Transfer in Bilaterally Symmetrical Flowers.” Oecologia 160: 667–674. 10.1007/s00442-009-1334-9. 19333624

[ece373221-bib-0037] Ushimaru, A. , and F. Hyodo . 2005. “Why Do Bilaterally Symmetrical Flowers Orient Vertically? Flower Orientation Influences Pollinator Landing Behaviour.” Evolutionary Ecology Research 7: 151–160.

[ece373221-bib-0038] Vasil, I. K. 1960. “Studies on Pollen Germination of Certain Cucurbitaceae.” American Journal of Botany 47: 239–247. 10.2307/2439602.

[ece373221-bib-0039] Wang, H. , S. Tie , D. Yu , Y.‐H. Guo , and C.‐F. Yang . 2014. “Change of Floral Orientation Within an Inflorescence Affects Pollinator Behavior and Pollination Efficiency in a Bee–Pollinated Plant, *Corydalis sheareri* .” PLoS One 9: e95381. 10.1371/journal.pone.0095381. 24743567 PMC3990675

[ece373221-bib-0040] Wang, H. , C. L. Xiao , R. W. Gituru , et al. 2014. “Change of Floral Orientation Affects Pollinator Diversity and Their Relative Importance in an Alpine Plant With Generalized Pollination System, *Geranium refractum* (Geraniaceae).” Plant Ecology 215: 1211–1219. 10.1007/s11258-014-0379-y.

[ece373221-bib-0041] Wang, Y. , L. H. Meng , Y. P. Yang , and Y. W. Duan . 2010. “Change in Floral Orientation in *Anisodus luridus* (Solanaceae) Protects Pollen Grains and Facilitates Development of Fertilized Ovules.” American Journal of Botany 97: 1618–1624. 10.3732/ajb.1000010. 21616797

[ece373221-bib-0042] Xia, M. Q. , Y. Liu , J. J. Liu , et al. 2022. “Out of the Himalaya– Hengduan Mountains: Phylogenomics, Biogeography and Diversification of *Polygonatum* Mill. (Asparagaceae) in the Northern Hemisphere.” Molecular Phylogenetics and Evolution 169: 107431. 10.1016/j.ympev.2022.107431. 35131418

[ece373221-bib-0043] Ye, Z. M. , X. F. Jin , Q. F. Wang , C. F. Yang , and D. W. Inouye . 2017. “Pollinators Shift to Nectar Robbers When Florivory Occurs, With Effects on Reproductive Success in *Iris bulleyana* (Iridaceae).” Plant Biology 19: 760–766. 10.1111/plb.12581. 28509436

[ece373221-bib-0044] Zhang, Z. J. , Y. F. Zhang , X. Y. Wang , et al. 2025. “Habitat Suitability of *Polygonatum cyrtonema* Based on MaxEnt Model in China.” Contemporary Problems of Ecology 18: 128–136. 10.1134/S199542552470094X.

[ece373221-bib-0045] Zhao, M. , H. Jia , J. Zhao , et al. 2025. “Response of Cultivation Suitability for *Polygonatum kingianum* to Climate Change in China.” Environmental Earth Sciences 84: 285. 10.1007/s12665-025-12304-2.

